# Proactive Tight Monitoring of Patients with Inflammatory Bowel Disease in Biological Therapy: A Controlled Clinical Study (TIME IT)

**DOI:** 10.3390/jcm15176900

**Published:** 2026-09-06

**Authors:** Katrine Risager Christensen, Osamah S. A. Al-Obaidi, Sine Buhl, Johan F. Ilvemark, Casper Steenholdt, Jørn Brynskov, Mark A. Ainsworth

**Affiliations:** 1 Department of Gastroenterology, Herlev and Gentofte, Copenhagen University Hospital, 2730 Herlev, Denmark; katrine.risager.christensen@regionh.dk (K.R.C.); sine_buhl@hotmail.com (S.B.); johan.ilvemark@gmail.com (J.F.I.); brynskov@icloud.com (J.B.); 2 Department of Medical Gastroenterology, Odense University Hospital, 5000 Odense, Denmark; osamah.saadi.abbas.al-obaidi@rsyd.dk (O.S.A.A.-O.); casper.steenholdt@rsyd.dk (C.S.); 3 Research Unit of Medical Gastroenterology, Department of Clinical Research, University of Southern Denmark, 5230 Odense, Denmark

**Keywords:** inflammatory bowel disease, treatment monitoring, mucosal healing, symptomatic control, treat-to-target

## Abstract

**Background/Objectives:** The combined goal of achieving both clinical and endoscopic remission has highlighted the need for strategies that support a treat-to-target approach in inflammatory bowel disease (IBD). A proactive monitoring strategy involves scheduled assessments regardless of symptoms to detect subclinical disease activity and enable timely treatment adjustments, whereas a reactive approach initiates evaluation only in response to clinical deterioration or objective signs of active disease. This study investigated whether proactive monitoring improves the achievement of treatment goals compared with a reactive strategy. **Methods:** In this prospective cohort study carried out between September 2020 and July 2022, patients with IBD receiving biologic therapy were managed according to a predefined proactive monitoring schedule and prospectively followed for one year. A control group, matched for age, sex, diagnosis, and treatment, was enrolled from another tertiary IBD center using a reactive monitoring strategy; their clinical course over one year was reconstructed retrospectively from medical records. All patients underwent colonoscopy assessment at one year. The primary outcome was treatment failure, defined as inadequate mucosal healing or symptom control, the need for intestinal surgery, or discontinuation of biologic therapy due to insufficient efficacy. **Results:** A total of 162 patients were included (91 managed proactively and 71 managed reactively). In the intention-to-treat population, treatment failure occurred in 67 patients (74%, 95% confidence interval (CI) 63–84%) in the intervention group and 60 patients (85%, 95% CI 75% to 94%) in the control group (difference 11%, CI −3% to 25%, *p* = 0.13). In the per-protocol analysis, 44 of 69 patients (64%, CI 50% to 78%) in the intervention group experienced treatment failure compared with 43 of 53 patients (81%, CI 60% to 93%) in the control group (difference 17%, CI −1% to 36%, *p* = 0.06). Patients in the proactive monitoring group underwent significantly more disease-monitoring assessments than those managed reactively. **Conclusions**: Proactive monitoring numerically improved combined clinical and endoscopic outcomes compared with reactive management, but this did not reach statistical significance and resulted in substantially greater healthcare resource utilization.

## 1. Introduction

Biologic therapy has improved disease control, reduced disease progression, and enhanced health-related quality of life (HRQOL) in patients with inflammatory bowel disease (IBD) [1,2,3]. Owing to the lifelong, unpredictable, relapsing–remitting nature of IBD, more than half of patients receiving biologic therapy will experience disease relapse [4]. Consequently, in an effort to alter the natural course of disease progression and reduce complication rates, treatment targets have evolved from achieving clinical remission alone to also including mucosal healing. Mucosal healing has been associated with a reduced risk of relapse and improved long-term outcomes, although the evidence remains limited [5,6,7].

Over the past few decades, rates of complications, bowel resections, and colectomies have declined in both ulcerative colitis (UC) and Crohn’s disease (CD) [8,9,10]. The treat-to-target strategy proposed by the Selecting Therapeutic Targets in IBD (STRIDE) initiative has become the predominant approach, aiming to achieve both clinical remission and mucosal healing (and even histologic healing) [11,12]. To facilitate a treat-to-target strategy and optimize treatment outcomes, it is important to distinguish between the two principal monitoring approaches: reactive monitoring and proactive monitoring.

In a proactive monitoring strategy, examinations are performed during both quiescent and active disease to detect early signs of evolving disease activity and enable timely therapeutic adjustments. In contrast, a reactive monitoring strategy initiates examinations and treatment modifications only in response to clinical deterioration and/or objective evidence of ongoing disease activity. Given the unpredictable course of both CD and UC, as well as the limited ability to predict treatment response, proactive monitoring is intuitively appealing. However, many questions regarding the optimal timing and use of monitoring tools remain unanswered [11,13,14].

The chronic nature of IBD, together with its increasing global incidence, places a growing burden on healthcare systems [15,16]. Therefore, it is important to identify monitoring strategies that provide optimal disease control while ensuring efficient use of healthcare resources and maintaining patient acceptability. Evidence supporting routine proactive monitoring in patients receiving biologic therapy remains limited and conflicting. Four studies have investigated the effects of implementing treat-to-target strategies based on proactive monitoring with predefined treatment algorithms in patients with CD. Two studies have demonstrated that proactive monitoring and treatment significantly improved outcomes [17,18]. In contrast, two other studies found no significant difference in outcomes between patients treated with a treat-to-target strategy and patients in the control group [19,20].

The aim of the present study was to investigate whether proactive monitoring reduces treatment failure compared with reactive monitoring.

## 2. Materials and Methods

### 2.1. Study Design

This was a prospective, non-randomized cohort study with a retrospectively assembled matched external control cohort. Patients with IBD receiving biologic therapy were prospectively enrolled at the tertiary IBD Clinic, Herlev and Gentofte Hospital, and managed according to a predefined proactive monitoring strategy. They were followed for 12 months, with clinical data and disease-monitoring assessments collected prospectively. A matched comparison cohort was identified at the tertiary IBD Clinic, Odense University Hospital, where patients receiving biologic therapy were managed according to a reactive monitoring strategy. Data for the comparison cohort, including the clinical course during the corresponding 12-month follow-up period, were obtained retrospectively from medical records. Patients were matched individually according to age, sex, IBD diagnosis, and biologic treatment (matching for smoking, disease duration, disease severity, and prior biologic exposure was not performed). All patients underwent colonoscopy assessment at one year. The study was conducted between September 2020 and July 2022. The two hospitals were comparable with respect to population size, demographic characteristics, biologic prescribing, access to surgery, and the number of specialists relative to the population served. The study only investigated the potential benefit of a monitoring regimen. The study did not include a fixed treatment algorithm. Treatment decisions in response to the results of the monitoring regimen were made at the discretion of the treating physician. Patients in the intervention group were followed prospectively for one year (52 weeks) according to a predefined proactive monitoring strategy. In this strategy, disease activity was assessed at fixed time points regardless of symptom status (Table 1). The proactive monitoring strategy has been described previously [21]. Monitoring included blood tests, therapeutic drug monitoring (TDM), fecal calprotectin measurements, disease activity indices, endoscopy, magnetic resonance imaging (MRI) when clinically relevant, gastrointestinal ultrasound (GIUS), and medical consultations. Disease activity was assessed using the Harvey–Bradshaw Index (HBI) for CD and the Simple Clinical Colitis Activity Index (SCCAI) for UC.

Monitoring schedules were adapted to individual biologic therapies to account for differences in administration route and dosing frequency. Patients were permitted to switch biologic therapy during the study period. Additional investigations beyond the predefined monitoring schedule could be performed at the discretion of the treating gastroenterologist when clinically indicated.

**Figure 1:**
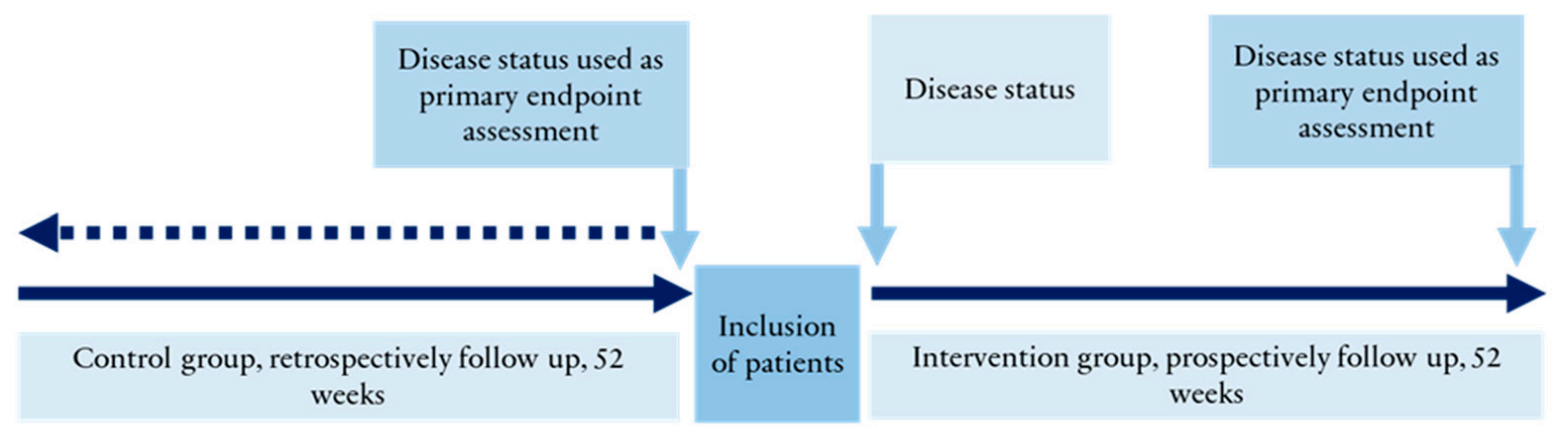
Study flow.

At baseline and at the end of the intervention period, disease status was evaluated using endoscopy and/or other relevant imaging modalities, including MRI when appropriate, together with disease activity indices and health-related quality of life (HRQOL) assessment using the Short Inflammatory Bowel Disease Questionnaire (SIBDQ) (Figure 1).

Patients in the control group were selected among patients who had been managed according to a reactive monitoring strategy, whereby clinical deterioration (as evaluated by the treating physician) or objective evidence of disease activity prompted further investigations and treatment adjustments at the discretion of the treating gastroenterologist. At inclusion, disease activity was assessed in a manner comparable to that used in the intervention group, including endoscopy and/or MRI, disease activity indices, and HRQOL assessment.

For patients in the control group, electronic medical records were reviewed retrospectively over a one-year period to collect information on investigations, including blood tests, fecal calprotectin measurements, MRI and other imaging modalities of the bowel or pelvis, GIUS, TDM, and medical consultations. Information regarding treatment modifications, including changes in biologic therapy and initiation of concomitant therapies, as well as surgery, symptoms, hospitalizations, and other clinically relevant outcomes, was also recorded.

### 2.2. Study Population

Patients were eligible for inclusion if they had an established diagnosis of CD or UC (based on established clinical, endoscopic, histologic, and radiologic criteria), were at least 18 years of age, and either had received biologic therapy for a minimum of three months or were initiating biologic treatment at study entry in the intervention group. In the control group, patients were required either to have received biologic therapy for at least three months or to have initiated biologic treatment between 1.0 and 1.25 years before inclusion. All participants provided written informed consent before enrollment.

Patients were excluded if they did not fulfill the safety criteria for initiation of biologic therapy, were pregnant or breastfeeding, or had comorbidities that would preclude participation in the proactive monitoring strategy at study entry.

Patients in the intervention group were recruited consecutively during routine visits to the IBD Clinic at Herlev and Gentofte Hospital between September 2020 and March 2021. Patients in the control group who fulfilled the eligibility criteria were matched to participants in the intervention group according to sex, age, diagnosis, and biologic therapy at inclusion and were recruited at Odense University Hospital between October 2020 and June 2022.

For both study groups, investigators remained blinded to disease activity status and previous medical-record information until written informed consent had been obtained.

### 2.3. Outcomes

The primary endpoint was treatment failure assessed after one year. Treatment failure was defined as insufficient endoscopic healing, insufficient symptomatic control, IBD-related surgery (e.g., bowel resection, fistulectomy), discontinuation of biologic therapy (because of loss of response or if biologic treatment was no longer considered a therapeutic option (e.g., patients who had failed multiple previous biologics or developed complications requiring non-medical intervention)), or initiation of systemic corticosteroid therapy during the study period.

Insufficient endoscopic healing was defined as an SES-CD score greater than 2 in CD [22,23] or a Mayo Endoscopic Subscore (MES) greater than 1 in UC [24]. Insufficient symptomatic control was defined as an HBI score greater than 5 [25,26] or an SCCAI score greater than 2 [27,28].

Secondary outcomes included differences between the intervention and control groups in endoscopic disease activity assessed by SES-CD and MES, clinical disease activity assessed by HBI and SCCAI, C-reactive protein (CRP), fecal calprotectin concentrations, and HRQOL. Differences in monitoring intensity were evaluated by comparing the number of blood tests, stool samples, medical consultations, disease activity assessments, GIUS examinations, and TDM measurements performed during follow-up. Clinical events occurring during the study period, including changes in biologic therapy and hospital admissions, were also recorded and compared between groups.

Patients who discontinued biologic therapy because of adverse events, switched to a small-molecule therapy, or relocated during follow-up were classified as dropouts.

### 2.4. Sample Size

The sample-size calculation was based on the assumption that the proportion of patients experiencing treatment failure would be lower in the intervention group than in the control group. Treatment failure in routine clinical practice was estimated to occur in approximately 37% of patients, while another study reported remission in approximately 10% of patients following a switch from infliximab to adalimumab [29,30].

Based on these observations and considering that patients with either CD or UC could switch among available biologic therapies, a clinically relevant difference of 20 percentage points in treatment failure rates between groups was assumed. Treatment failure rates were estimated to be 20% in the intervention group and 40% in the control group. Using a significance level (α) of 0.05 and a power corresponding to β = 0.20, a total sample size of 162 patients, including 81 patients in each group, was calculated.

The planned sample size was achieved in the intervention group. However, recruitment of control-group participants was terminated after inclusion of 72 of the intended 81 patients because of slow enrollment related to restrictions associated with the COVID-19 pandemic.

### 2.5. Statistical Analyses

The baseline demographic and characteristics of included patients were summarized using descriptive statistics (percentages for discrete and categorical variables and median with interquartile range (IQR) or mean with standard deviation (SD) for continuous variables). Categorical variables were compared between the intervention and control groups using the chi-squared test. Continuous variables were compared using the Wilcoxon rank-sum test for non-normally distributed data and the independent two-sample *t*-test for normally distributed data. The primary binary endpoint was analyzed both in the intention-to-treat (ITT) population (all included patients) and per-protocol (PP) population (excluding dropouts and patients with missing end-of-follow-up disease status assessment). Missing data (including dropouts) in the ITT population were implemented as “non-responder imputation” (treatment failure). To evaluate the resilience of the primary outcome, a subgroup analysis was performed. Sensitivity analyses using different methods for handling missing data were also performed. A chi-squared test was performed to analyze the primary endpoint. Two-sided *p*-values < 0.05 were considered statistically significant. In addition, odds ratios (ORs) with 95% confidence intervals (95% CIs) were calculated with univariate and multivariable logistic regression with treatment failure as the dependent variable and intervention vs. control group, diagnosis, treatment administration (infusion versus injection), concomitant immunosuppressive therapy, and course of treatment (induction vs. maintenance) as independent variables. Statistical analyses were performed in R version 4.3.2 (31 October 2023ucrt) [31].

## 3. Results

### 3.1. Baseline Characteristics

**Figure 2:**
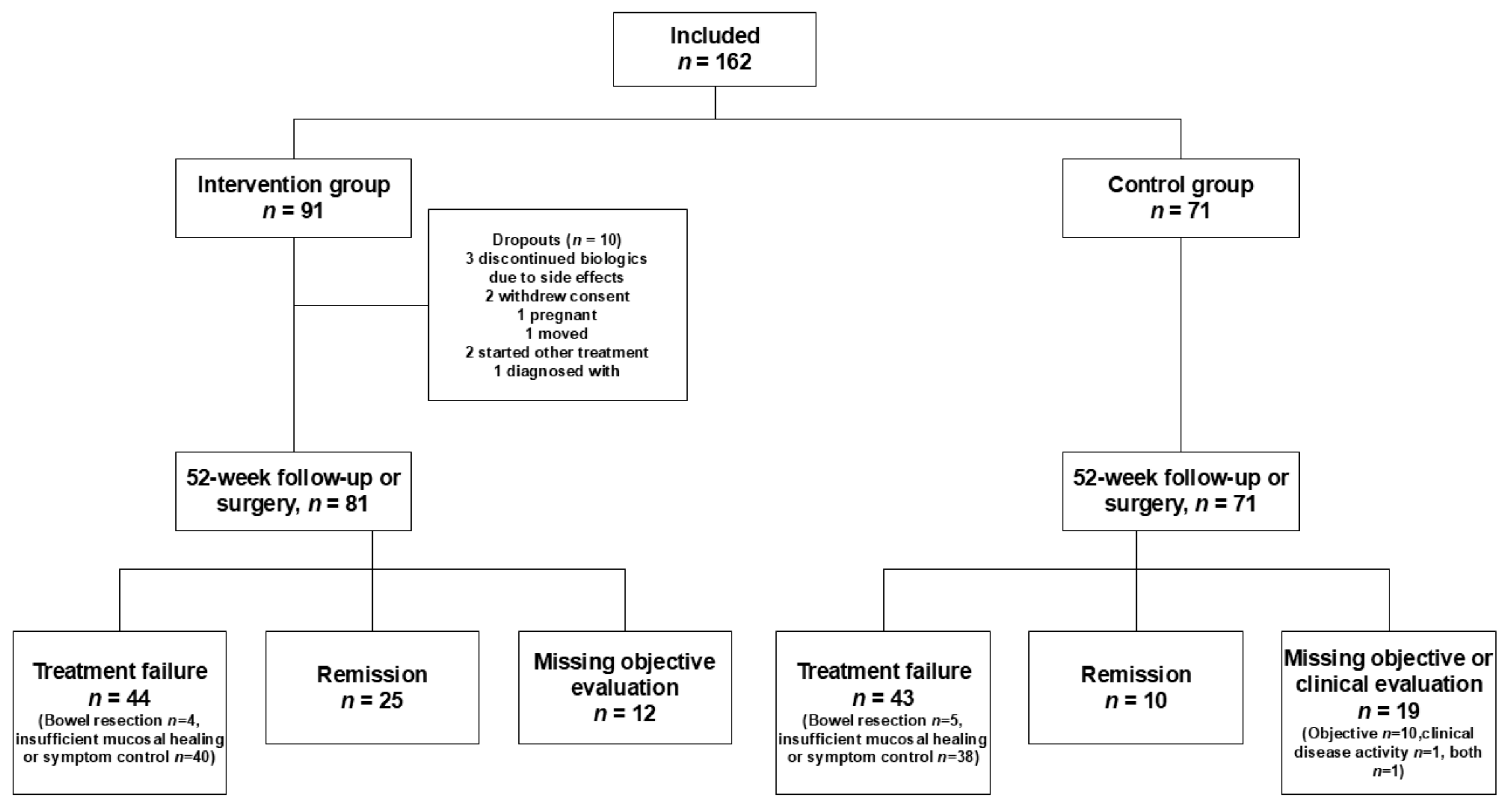
Patient flowchart.

A total of 162 patients were included in the study, comprising 91 patients in the intervention group and 71 patients in the control group (Figure 2). The median follow-up duration was 369 days (IQR 353–389) in the intervention group and 365 days (IQR 346–381) in the control group. Baseline characteristics are summarized in Table 2.

Overall, the two groups were broadly comparable with respect to age, sex, diagnosis, disease duration, type of biologic therapy, body weight, inflammatory markers, fecal calprotectin concentrations, and previous exposure to biologic therapy. However, several clinically relevant differences were observed at baseline. Patients in the intervention group had received their current biologic therapy for a significantly shorter period than patients in the control group (median 6 months [IQR 0–30] vs. 36 months [IQR 11.8–48.5], *p* < 0.001). Accordingly, a greater proportion of patients in the intervention group were receiving induction therapy at study entry (45.1% vs. 12.7%, *p* < 0.001).

The use of concomitant medication also differed between groups. Patients in the intervention group were less likely to receive biologic monotherapy and more likely to receive concomitant treatment than patients in the control group (*p* = 0.016). In addition, smoking status differed significantly between groups (*p* = 0.001), although the proportions of current smokers were relatively low in both cohorts.

Among patients with ulcerative colitis, baseline disease activity assessed by SCCAI was higher in the intervention group than in the control group (median 5 [IQR 3–8] vs. 1 [IQR 1–4], *p* = 0.001). Similarly, mean serum albumin concentrations were lower in the intervention group (39.4 ± 3.8 g/L vs. 43.2 ± 5.8 g/L, *p* < 0.001). In contrast, baseline HBI scores, CRP concentrations, fecal calprotectin levels, disease duration, and previous biologic exposure did not differ significantly between groups.

### 3.2. Primary Endpoint: Treatment Failure

**Figure 3:**
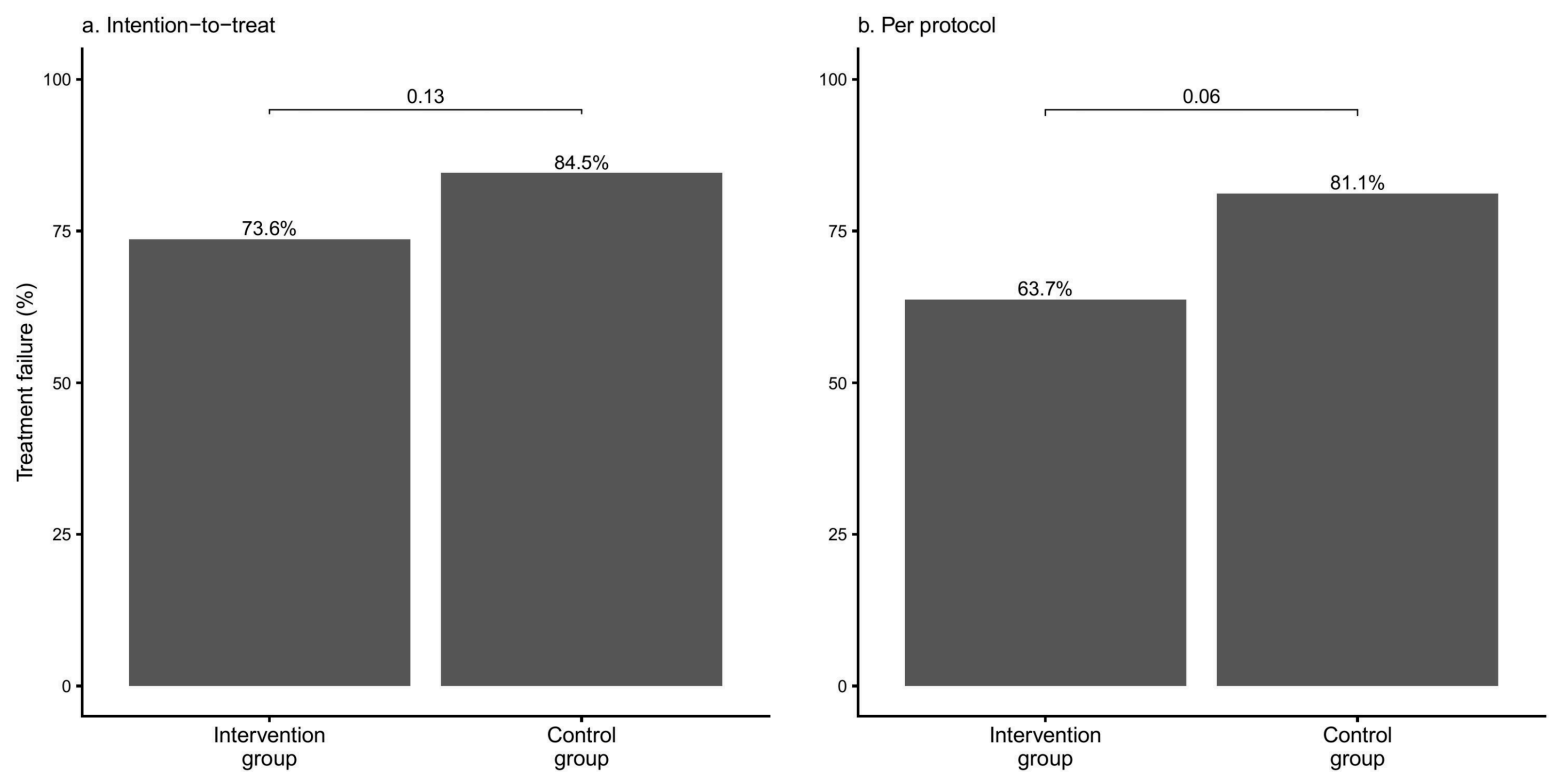
Primary endpoint: treatment failure.

After 52 weeks of follow-up, treatment failure occurred in 67 of 91 patients (74%, 95% confidence interval (CI) 63% to 84%) in the intervention group and in 60 of 71 patients (85%, CI 75% to 94%) in the control group in the intention-to-treat (ITT) analysis, corresponding to a non-significant difference between groups of 11% (CI −3% to 25% *p* = 0.13) (Figure 3a).

In the per-protocol (PP) analysis, treatment failure was observed in 44 of 69 patients (64%, CI 50% to 78%) in the intervention group compared with 43 of 53 patients (81%, CI 70% to 93%) in the control group. Although this difference (17%, CI −1% to 36%) favored the proactive monitoring strategy, statistical significance was not achieved (*p* = 0.06) (Figure 3b).

Complete objective and clinical disease assessments, including endoscopic and/or radiological evaluations together with symptom-based disease activity measures, were available for 69 patients (76%) in the intervention group and 53 patients (75%) in the control group. Sensitivity analyses using alternative methods for handling missing data yielded results consistent with the primary analysis, supporting the robustness of the findings (Supplementary File, Supplementary Tables S1 and S2).

#### 3.2.1. Risk of Treatment Failure

Univariate and multivariable logistic regression analyses were performed to identify factors associated with treatment failure. In the ITT population, no significant associations were observed between treatment failure and study group (intervention vs. control), diagnosis (CD vs. UC), route of biologic administration (injection vs. infusion), treatment phase (induction vs. maintenance), or concomitant immunosuppressive therapy (Supplementary File, Supplementary Table S3).

In the PP population, univariate analysis demonstrated a significantly lower risk of treatment failure among patients managed with the proactive monitoring strategy compared with those managed using a reactive approach (OR 0.41, 95% CI 0.18–0.95; *p* = 0.04), consistent with the trend observed in the primary PP analysis. However, this association was no longer statistically significant after adjustment for potential confounders in the multivariable model. No other variables were significantly associated with treatment failure in either the univariate or multivariable analyses (Supplementary File, Supplementary Table S4).

#### 3.2.2. Subgroup Analysis of Treatment Failure

When the primary endpoint was restricted to objective measures of disease activity, including endoscopic findings, MRI findings, and IBD-related surgery, while excluding clinical disease activity indices, treatment failure was observed in 53 of 91 patients (58%, CI 45% to 72%) in the intervention group and 43 of 71 patients (61%, CI 46% to 75%) in the control group in the ITT population (*p* = 0.89). Similarly, in the PP population, treatment failure occurred in 31 of 69 patients (45%, CI 27% to 62%) in the intervention group and 32 of 53 patients (60%, CI 43% to 77%) in the control group, with no statistically significant difference between groups (*p* = 0.33).

Additional subgroup analyses were performed in patients receiving maintenance therapy at study entry. In the ITT population, treatment failure occurred in 39 of 50 patients (78%, CI 65% to 91%) in the intervention group and 51 of 62 patients (82%, CI 72% to 93%) in the control group (*p* = 0.75). Corresponding rates in the PP population were 31 of 42 patients (74%, CI 58% to 89%) and 35 of 45 patients (78%, CI 64% to 92%), respectively (*p* = 0.85).

In the maintenance-therapy subgroup, neither univariate nor multivariable logistic regression analyses identified significant predictors of treatment failure in the ITT population. In the PP population, univariate analysis demonstrated a lower risk of treatment failure among patients receiving biologic therapy administered by infusion compared with injection (OR 0.39, 95% CI 0.21–0.66; *p* = 0.001). However, no other variables were significantly associated with treatment failure, and this association was not confirmed in the multivariable analysis (Supplementary File, Supplementary Table S5).

### 3.3. Secondary Outcomes

#### 3.3.1. Disease Activity and Quality of Life

At the end of follow-up, no significant differences in endoscopic disease activity were observed between the intervention and control groups. Among patients with CD and available endoscopic assessments, an SES-CD score > 2 was recorded in 20 of 38 patients (53%, CI 31% to 75%) in the intervention group and 21 of 42 patients (50%, CI 29% to 71%) in the control group (*p* = 0.99). Similarly, median SES-CD scores did not differ significantly between groups (2 [IQR 0–6.75] vs. 3 [IQR 0–5], *p* = 0.77).

Among patients with UC and available endoscopic assessments, an MES > 1 was observed in 8 of 29 patients (28%, CI −3% to 59%) in the intervention group and 2 of 8 patients (25%, CI −35% to 85%) in the control group (*p* = 1.00). Median MES scores were likewise comparable between groups (0 [IQR 0–2] vs. 1 [IQR 1–1.25], *p* = 0.13). With regard to clinical disease activity, patients with CD in the intervention group had significantly lower HBI scores at the end of follow-up than those in the control group (median 3 [IQR 1–5] vs. 5 [IQR 2.5–7], *p* = 0.02). However, the proportion of patients with an HBI score > 5 did not differ significantly between groups (22.9% vs. 34.8%, *p* = 0.30). Among patients with UC, no significant differences were observed in either SCCAI scores or the proportion of patients with an SCCAI score > 2. The median SCCAI score was 2 (IQR 1–3.5) in the intervention group and 3 (IQR 1–4) in the control group (*p* = 0.46), while SCCAI scores > 2 were observed in 44% and 55.6% of patients, respectively (*p* = 0.57).

No significant differences were observed between groups in inflammatory biomarkers. Mean CRP concentrations were 2 mg/L (SD 4.45) in the intervention group and 6 mg/L (SD 16.5) in the control group (*p* = 0.29). Likewise, median fecal calprotectin concentrations were comparable between groups (74.5 μg/g [IQR 21.25–194.5] vs. 95 μg/g [IQR 29–556], *p* = 0.36).

Health-related quality of life, assessed using the SIBDQ, was similar in the two groups at the end of follow-up. Median SIBDQ scores were 54 (IQR 47–60.5) in the intervention group and 51.5 (IQR 46.25–61.75) in the control group (*p* = 0.70).

#### 3.3.2. Number of Examinations and Outcomes During the One-Year Study Period

**Figure 4:**
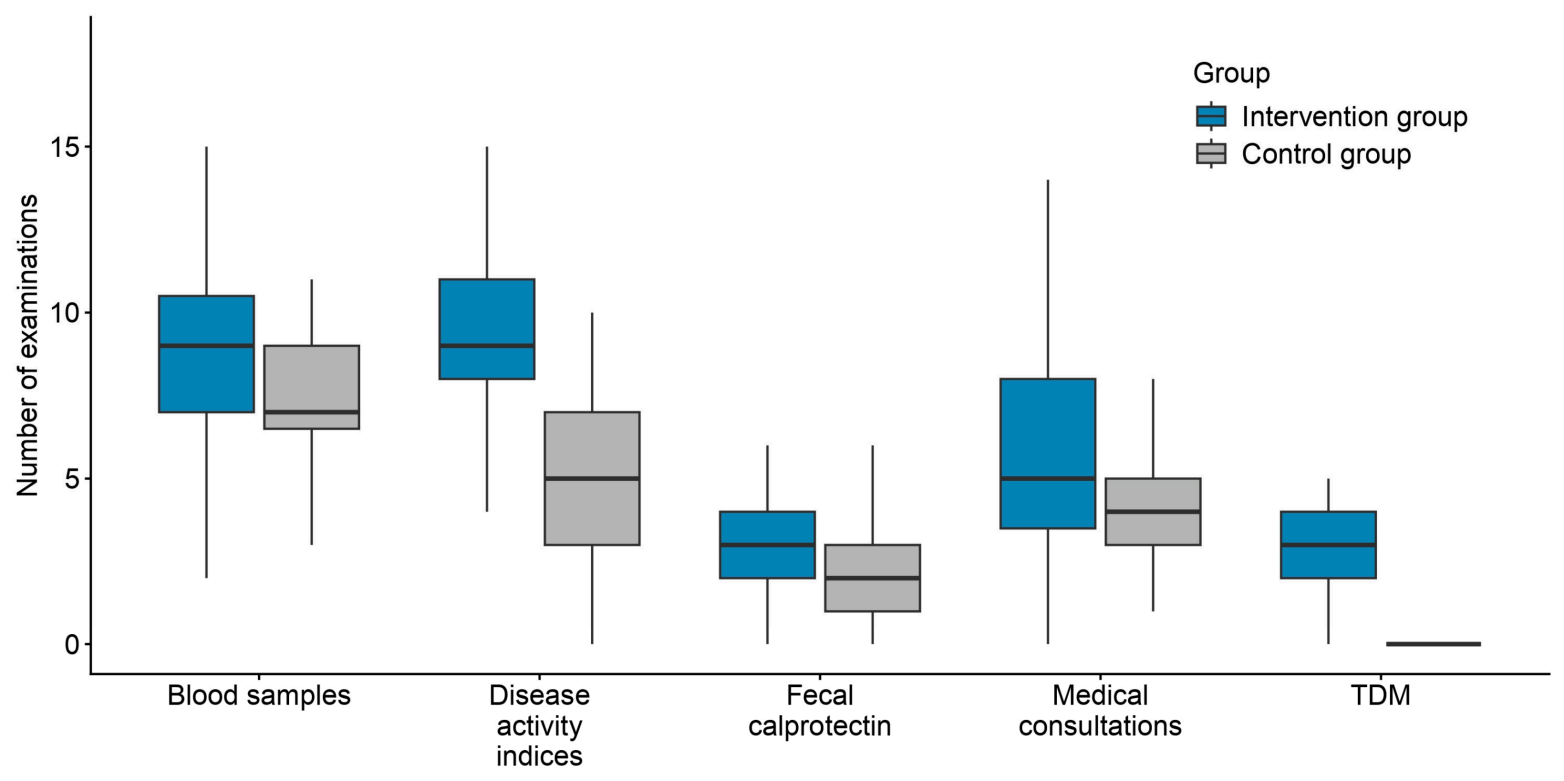
Number of examinations and tools performed during follow-up in the intervention group and the control group. TDM: therapeutic drug monitoring. Horizontal lines represent medians, and boxes indicate interquartile ranges.

As expected, patients managed according to the proactive monitoring strategy underwent substantially more disease-monitoring assessments during follow-up than patients managed using a reactive approach (Figure 4). The intervention group had significantly higher numbers of blood tests, therapeutic drug monitoring measurements, medical consultations, disease activity assessments, and fecal calprotectin measurements than the control group (all *p* ≤ 0.03). Therapeutic drug monitoring showed the largest difference between groups, with a median of three assessments (IQR 2–4) performed in the intervention group compared with none in the control group (*p* < 0.001). Similarly, the frequency of disease activity assessments was almost twofold higher in the intervention group than in the control group (median 9 [IQR 8–11] vs. 5 [IQR 3–7], *p* < 0.001).

Gastrointestinal ultrasound was routinely incorporated into the proactive monitoring strategy and was available only at the IBD Clinic, Herlev Hospital. Patients in the intervention group underwent a median of three GIUS examinations (IQR 2–4) during the study period.

Treatment modifications were more frequent among patients managed with proactive monitoring. During follow-up, 24 patients (26.4%) in the intervention group changed biologic therapy compared with 7 patients (9.8%) in the control group (*p* = 0.005).

Hospital admission rates did not differ significantly between groups. Eleven patients (12%) in the intervention group and 13 patients (18.3%) in the control group were hospitalized because of IBD-related disease activity and/or complications during follow-up (treatment difference 6%, CI −22% to 35%, *p* = 0.18).

## 4. Discussion

In this controlled clinical study, proactive monitoring did not significantly reduce treatment failure compared with a reactive monitoring strategy in patients with IBD receiving biologic therapy. Although numerically lower rates of treatment failure were observed in the intervention group, particularly in the per-protocol analysis, these differences did not reach statistical significance. The proactive monitoring strategy was, however, associated with substantially greater use of healthcare resources, including more frequent investigations, monitoring procedures, and treatment modifications.

To our knowledge, this is the first study to evaluate a proactive monitoring strategy in an unselected, real-world IBD population without the use of a predefined treatment algorithm. Overall, treatment failure rates were high in both study groups. In the ITT analysis, treatment failure occurred in approximately three-quarters of patients in the intervention group and more than four-fifths of patients in the control group. Similar findings were observed in the PP analysis. When treatment failure was assessed using objective outcomes only, including endoscopic findings, imaging results, and IBD-related surgery, failure rates remained high and were comparable between groups. Subgroup analyses did not materially alter these findings. Previous studies evaluating treat-to-target approaches have similarly reported relatively low rates of combined clinical and endoscopic remission after one year of treatment [17,18,19,20,32,33,34,35].

The high proportion of patients failing to achieve the composite treatment target may reflect the characteristics of the study population. Patients had a median disease duration of approximately 11 years, and more than half had been exposed to biologic therapy before initiation of their current treatment. Several studies have demonstrated that earlier introduction of biologic therapy and treatment in biologic-naïve patients are associated with improved clinical and endoscopic outcomes [17,35]. Consequently, the potential benefit of proactive monitoring may be more difficult to demonstrate in a population with longstanding disease and previous biologic exposure. It is also possible that a longer follow-up period would be required to detect clinically meaningful differences between monitoring strategies.

Although combined clinical and endoscopic remission is increasingly regarded as the preferred therapeutic target in IBD, there is no universally accepted definition of remission. In the present study, limited residual symptoms and mild endoscopic activity were permitted within the definition of remission (HBI ≤ 5, SCCAI ≤ 2, SES-CD ≤ 2, and MES ≤ 1), consistent with commonly applied thresholds in clinical research and practice [23,28,36,37]. Complete resolution of symptoms and normalization of mucosal appearance may not be achievable in all patients, particularly those with longstanding disease. Nevertheless, more stringent treatment targets may ultimately be required to optimize long-term outcomes. In ulcerative colitis, increasing attention has been directed toward histological healing as an additional treatment goal, whereas evidence supporting a similar approach in CD remains limited [38].

In contrast to the CALM study, which demonstrated improved outcomes using a tight-control strategy based on predefined treatment-escalation criteria [17], the present study did not incorporate a formal treatment algorithm. The proactive monitoring strategy provided clinicians with more clinical information and objective disease assessments; however, treatment decisions remained at the discretion of individual physicians. Consequently, responses to evidence of ongoing disease activity may have varied between clinicians. Some physicians may have intensified treatment promptly, whereas others may have adopted a more conservative approach. This variation may have reduced the potential impact of proactive monitoring. Furthermore, elements of structured follow-up were also present in the control group, including regularly performed laboratory investigations, fecal calprotectin measurements, and outpatient consultations, which may have narrowed the distinction between the two monitoring strategies.

Interestingly, subgroup analyses suggested that patients receiving infusion-based biologic therapy experienced lower rates of treatment failure than patients receiving self-administered injectable therapies. Although this association was not confirmed in multivariable analyses, the finding may reflect the closer clinical contact associated with infusion-based treatment. Regular attendance at an IBD clinic for infusions provides repeated opportunities for assessment by nurses and physicians and may function as a form of informal tight monitoring. Such interactions could potentially reduce differences between proactive and reactive management strategies.

Despite the absence of significant differences in clinical outcomes, the intervention clearly increased healthcare utilization. Patients in the proactive monitoring group underwent substantially more blood tests, fecal calprotectin measurements, disease activity assessments, therapeutic drug monitoring procedures, outpatient consultations, and imaging investigations. They were also more likely to change biologic therapy during follow-up. These findings demonstrate that proactive monitoring can be implemented in routine clinical practice but at the cost of considerable additional resource utilization. Whether such an approach is cost-effective remains uncertain.

Optimizing disease monitoring in IBD remains challenging because of the highly heterogeneous disease course and the limited ability to predict individual treatment response. Furthermore, important uncertainties remain regarding the optimal timing, frequency, and interpretation of many monitoring tools [11,13,14,39]. The time required to achieve mucosal healing and biomarker normalization differs according to disease phenotype, disease severity, and treatment modality. Consequently, inappropriate timing of disease assessment may underestimate treatment response. Although repeated endoscopic evaluation may provide the most accurate assessment of mucosal healing, patient acceptance is often limited, and the procedures are associated with costs and procedural risks. Non-invasive monitoring tools, including fecal calprotectin and GIUS, therefore represent attractive alternatives. Fecal calprotectin has been shown to predict sustained response in selected patient populations [40], but adherence to repeated stool sampling may be suboptimal [21,41]. GIUS is non-invasive, well accepted by patients, and increasingly recognized as a valuable tool for disease monitoring in selected clinical settings [41,42]. Further studies are needed to determine the most effective combination of monitoring modalities within a treat-to-target framework.

Several limitations should be acknowledged. First, complete endoscopic and imaging assessments were not available for all patients at the end of follow-up, partly because of disruptions related to the COVID-19 pandemic. Second, the study population was heterogeneous with respect to disease type, disease location, disease severity, biologic treatment, and treatment phase, which may have increased variability and reduced the ability to detect subgroup-specific effects. Third, the non-randomized study design with an external control group introduces the possibility of residual confounding despite matching for age, sex, diagnosis, and biologic therapy. Baseline differences between groups remained, particularly regarding treatment duration, treatment phase, and disease activity. In addition, inclusion of patients who had undergone endoscopic assessment up to two months before enrollment may have introduced selection bias and confounding by indication. Furthermore, the study was carried out in part during the COVID pandemic. While IBD care was generally unchanged during the pandemic, it cannot be ruled out that this could have influenced the results. Finally, recruitment of the control group was slower than anticipated, resulting in a modest reduction in statistical power relative to the original sample-size calculation. Failure to demonstrate a statistically significant effect of proactive monitoring could have been secondary to this lack of power, as suggested by the borderline significant difference in the PP analyses (*p* = 0.06).

## 5. Conclusions

Proactive monitoring numerically improved combined clinical and endoscopic outcomes compared with reactive disease monitoring in patients with IBD receiving biologic therapy. However, the difference did not attain statistical significance, and proactive monitoring was associated with substantially greater healthcare resource utilization, including more frequent investigations and treatment modifications. Overall, the findings suggest that proactive monitoring alone may be insufficient to improve outcomes in routine clinical practice unless combined with clearly defined therapeutic decision-making pathways. Future studies should evaluate whether monitoring strategies integrated with standardized treatment algorithms, risk stratification, and cost-effectiveness analyses can improve long-term outcomes while maintaining efficient use of healthcare resources.

## Figures and Tables

**Table 1:** Example of proactive monitoring strategy for a drug administered every 8 weeks.

Events	Visit I	Visit II	Visit III	Visit IV	Visit V	Visit VI	Visit VII
Weeks	0	8	16	24	32	40	48
Routine blood samples ^a^	X	X	X	X	X	X	X
Vitamin and mineral status (B, D, and iron)	X			X			X
Therapeutic drug monitoring							
Biologic therapy ^b^	●	□	●	□	●	□	●
Thiopurines ^c^	●		□	●		□	●
Fecal calprotectin	●	□	●	□	●	□	●
Disease activity index ^d^	X	X	X	X	X	X	X
Endoscopy	●					□	●
Magnetic resonance imaging (Crohn’s disease if relevant)/other imaging modalities	●					□	●
Ultrasound examination of the bowel	X			X			X
Medical consultations	X			X			X
Administration of medicine (nurse)	X	X	X	X	X	X	X

Example using infusion as route of administration and drug frequency of 8 weeks. ^a^ Hemoglobin, complete blood count, creatinine, potassium, sodium, albumin, alkaline phosphatase, alanine aminotransferase, bilirubin, C-reactive protein (CRP), international normalized ratio, vitamin D, and iron. ^b^ Drug concentration level in blood + drug–antibodies measured if low drug concentration. ^c^ Erythrocyte 6-thioguanine nucleotide (6-TGN) and methylated metabolites (MeMPs). ^d^ Disease activity indices: Harvey–Bradshaw for Crohn’s disease and Simple Clinical Colitis Activity Index for ulcerative colitis. X: to be executed on visit (same-day results); ●: check results; □: book/plan for next visit.

**Table 2:** Baseline characteristics.

Characteristics	Intervention Group	Control Group	*p*
**No. of patients**	91	71	
**Age, years (mean (SD))**	43.32 (14.82)	41.13 (14.42)	0.346
**Gender, No. (%)**			0.763
Female	52 (57.1)	38 (53.5)	
Male	39 (42.9)	33 (46.5)	
**Diagnosis, No. (%)**			0.354
Crohn’s disease	53 (58.2)	48 (67.6)	
Ulcerative colitis	37 (40.7)	23 (32.4)	
**Disease duration, years (median [interquartile range IQR])**	11.50 [6–18.75]	11 [8–18]	0.350
**Treatment, No. (%)**			0.133
Infliximab	53 (58.9)	30 (42.2)	
Adalimumab	10 (11.1)	8 (11.2)	
Golimumab	2 (2.2)	5 (7)	
Vedolizumab	18 (20.0)	17 (23.9)	
Ustekinumab	7 (7.8)	11 (15.4)	
**Duration of baseline biologic therapy, months (median [IQR])**	6 [0–30]	36 [11.75–48.5]	**<0.001**
**Course of treatment, No. (%)**			**<0.001**
Induction	41 (45.1)	9 (12.7)	
Maintenance	50 (54.9)	62 (87.3)	
**Concomitant treatment, No. (%)**			**0.016**
None	49 (53.2)	48 (67.6)	
Azathiopurine	18 (19.8)	11 (15.5)	
Azathiopurine and allopurinol	5 (5.43)	2 (2.8)	
Mercaptopurine	3 (3.26)	1 (1.4)	
Mercaptopurine and allopurinol	1 (1.1)	0 (0)	
Methotrexate	2 (2.17)	1 (1.4)	
Mesalazine	27 (29.3)	6 (8.5)	
Corticosteroids	7 (7.6)	4 (4.9)	
Topical corticosteroid	1 (1.1)	NA	
**Weight, kilograms (mean (SD))**	77.34 (14.67)	83.85 (21.66)	0.059
**Smoking, No. (%)**			**0.001**
Non-smoker	44 (60.3)	21 (44.7)	
Smoker	24 (32.9)	10 (21.3)	
Previous smoker	5 (6.8)	16 (34.0)	
**SCCAI, score (median [IQR])**	5 [3–8]	1 [1–4]	**0.001**
**HBI, score (median [IQR])**	4 [1–8]	3 [1–4.50]	0.215
**C-reactive protein, mg/L (median [IQR])**	0 [0–5]	0 [0–6.80]	0.401
**Hemoglobin, mmol/L (mean (SD))**	8.51 (0.93)	8.53 (0.99)	0.810
**Albumin, g/L (mean (SD))**	39.40 (3.78)	43.17 (5.76)	**<0.001**
**Fecal calprotectin (median μg/g [IQR])**	271 [44–1120]	304 [60.25–715.25]	0.835
**Previous number of biologics, No. patients (%)**			0.604
0	42 (46.2)	31 (43.7)	
1	32 (35.2)	23 (32.4)	
2	12 (13.2)	9 (12.7)	
3	2 (2.2)	5 (7.0)	
4	3 (3.3)	2 (2.8)	
5	0 (0.0)	1 (1.4)	
**Previous surgery, No. (median [IQR])**	0 [0–0]	0 [0–1]	**NA**

## Data Availability

The data presented in this study are available upon request from the corresponding author due to Danish Data Protection Regulations.

## Supplementary Materials

The following supporting information can be downloaded at https://www.mdpi.com/article/10.3390/jcm15176900/s1.

## Abbreviations

The following abbreviations are used in this manuscript:

CD	Crohn’s Disease
CI	Confidence Interval
CRP	C-Reactive Protein
GIUS	Gastrointestinal Ultrasound (ultrasound examination of the bowel)
HBI	Harvey–Bradshaw Index (disease activity index for Crohn’s disease)
HRQOL	Health-Related Quality of Life
IBD	Inflammatory Bowel Disease
IQR	Interquartile Range
ITT	Intention to Treat (analysis population)
MeMPs	Methylated Metabolites (of thiopurines)
MES	Mayo Endoscopic Subscore (for ulcerative colitis)
MRI	Magnetic Resonance Imaging
OR	Odds Ratio
PP	Per Protocol (analysis population)
SCCAI	Simple Clinical Colitis Activity Index (disease activity index for ulcerative colitis)
SD	Standard Deviation
SES-CD	Simple Endoscopic Score for Crohn’s Disease
SIBDQ	Short Inflammatory Bowel Disease Questionnaire (measure of HRQOL)
STRIDE	Selecting Therapeutic Targets in IBD (initiative)
TDM	Therapeutic Drug Monitoring
6-TGN	6-Thioguanine Nucleotide (metabolite of thiopurines)
UC	Ulcerative Colitis
