# Supplementary material for: Proactive Tight Monitoring of Patients with Inflammatory Bowel Disease in Biological Therapy: A Controlled Clinical Study (TIME IT)
Source: J Clin Med. 2026 Sep 6;15(17):6900. doi: 10.3390/jcm15176900 (PMC13565967; doi:10.3390/jcm15176900)
Supplement: Supplementary file 1 [file jcm-15-06900-s001.zip › jcm-4455991-supplementary.pdf]

**SUPPLEMENTARY MATERIAL**

# Proactive tight monitoring of patients with inflammatory bowel disease in biological therapy: A controlled clinical study (TIME IT)

Katrine Risager Christensen (MD, PhD)[1,2], Osamah S. Al-Obaidi (MD)[2], Sine Buhl (MD, PhD)[1], Johan F. Ilvemark (MD, PhD)[1], Casper Steenholdt (MD, PhD, DMSc)[2,3], Jørn Brynskov (MD, DMSc) [1], Mark A. Ainsworth (MD, PhD, DMSc) [2, 3]

[1] Copenhagen University Hospital, Herlev and Gentofte, Dept. of Gastroenterology, Denmark

[2] Department of Medical Gastroenterology, Odense University Hospital, Denmark

[3] Research Unit of Medical Gastroenterology, Department of Clinical Research, University of Southern Denmark, Odense, Denmark

**Corresponding author**

Katrine Risager Christensen

Herlev and Gentofte Hospital

Dept. of Gastroenterology

Borgmester Ib Juuls Vej 1

2730 Herlev

e-mail: Katrine.risager.christensen@regionh.dk

mobile: 0045 29382334

## Sensitivity analyzes

Intention to treat population, imputation of missing values

**Table S1: Scenario # 1**

<table>
<tr><td colspan="2" rowspan="2">Imputation model</td><td colspan="2">Objective evaluation (including surgery)</td></tr>
<tr><td>Available</td><td>Not available</td></tr>
<tr><td rowspan="2">Disease activity indices (Harvey-Bradshaw Index/Simple Clinical Colitis Activity Indices)</td><td>Available</td><td>Both treatment failure = treatment failure, one remission and one treatment failure = treatment failure, both remission = remission</td><td>Disease activity index is decisive</td></tr>
<tr><td>Not available</td><td>Objective investigation is decisive</td><td>Treatment failure</td></tr>
</table>

Distribution of patients following imputation scenario #1

| (No patients) | Failure | Remission |
|---|---|---|
| Intervention group | 60 | 31 |
| control group | 52 | 19 |

P-value = 0.408

**Table S2: Scenario # 2**

<table>
<tr><td colspan="2" rowspan="2">Imputation model</td><td colspan="2">Objective evaluation (including surgery)</td></tr>
<tr><td>Available</td><td>Not available</td></tr>
<tr><td rowspan="2">Disease activity indices (Harvey-Bradshaw Index/Simple Clinical Colitis Activity Indices)</td><td>Available</td><td>Both treatment failure = treatment failure, one remission and one treatment failure = treatment failure, both remission = remission</td><td>Disease activity index is decisive</td></tr>
<tr><td>Not available</td><td>Objective investigation is decisive</td><td>Remission</td></tr>
</table>

Distribution of patients following scenario # 2

| (No. of patients) | Failure | Remission |
|---|---|---|
| Intervention group | 52 | 39 |
| control group | 47 | 24 |

P-value = 0.3122

## Logistic regression

Univariant analysis and multivariant analyses performed with logistic regression. OR: odds ratio, 95% CI (95 % confidential intervals). Treatment failure as dependent variable and [a]intervention vs control group, [b]diagnosis (Crohn's disease vs. ulcerative colitis), [c]treatment administration (injection vs. infusion), and [d]concomitant therapy (additional immunosuppressive yes/no) [e]treatment course (induction vs. maintenance) as independent variables

**Table S3: Intention to treat population**

**Intention to treat, logistic regression, univariate analyzes**

| Variables | OR | 95% CI | p-value |
|---|---|---|---|
| Group[a] | 0.51 | 0.23 – 1.13 | 0.0983 |
| Diagnosis[b] | 1.13 | 0.53 – 2.44 | 0.7460 |
| Treatment administration[c] | 0.75 | 0.3 – 1.92 | 0.5500 |
| Concomitant therapy[d] | 0.72 | 0.3 – 1.73 | 0.4640 |
| Treatment course[e] | 0.70 | 0.32 – 1.53 | 0.3650 |

**Intention to treat, logistic regression, multivariate analyzes**

| Variables | OR | 95% CI | p-value |
|---|---|---|---|
| Group[a] | 0.52 | 0.21-1.23 | 0.144 |
| Diagnosis[b] | 1.01 | 0.44-2.23 | 0.985 |
| Treatment administration[c] | 0.66 | 0.24-1.65 | 0.397 |
| Concomitant therapy[d] | 0.61 | 0.23-1.46 | 0.287 |
| Treatment course[e] | 0.88 | 0.37-2.13 | 0.770 |

**Table S4: Per protocol population**

**Per protocol, logistic regression, univariate analyzes**

| Variable | OR | 95% CI | p-value |
|---|---|---|---|
| Group[a] | 0.41 | 0.18 – 0.95 | 0.0383 |
| Diagnosis[b] | 0.71 | 0.27 – 1.88 | 0.4900 |
| Treatment administration[c] | 0.48 | 0.21 – 1.1 | 0.0828 |
| Concomitant therapy[d] | 1.48 | 0.66 – 3.35 | 0.3440 |
| Treatment course[e] | 0.69 | 0.29 – 1.67 | 0.4120 |

**Per protocol, logistic regression, multivariate analyzes**

| **Variable** | OR | 95% CI | p-value |
|---|---|---|---|
| Group[a] | 0.45 | 0.18-1.08 | 0.0803 |
| Diagnosis[b] | 1.33 | 0.56-3.14 | 0.5130 |
| Treatment administration[c] | 0.59 | 0.20-1.56 | 0.3030 |
| Concomitant therapy[d] | 0.56 | 0.21-1.37 | 0.2190 |
| Treatment course[e] | 0.56 | 0.22-1.39 | 0.2040 |

**Table S5: Logistic regression, patients in maintenance therapy**

**Intention to treat, logistic regression, univariate**

| **Variables** | OR | 95% CI | p-value |
|---|---|---|---|
| Group[a] | 0.8 | 0.3- 1.96 | 0.57 |
| Diagnosis[b] | 1.27 | 0.46-3.31 | 0.64 |
| Treatment administration[c] | 0.59 | 0.15- 1.78 | 0.38 |
| Concomitant therapy[d] | 0.86 | 0.26-2.45 | 0.784 |

**Intention to treat, logistic regression, multivariate**

| Variables | OR | 95% CI | p-value |
|---|---|---|---|
| Group[a] | 0.65 | 0.25-1.67 | 0.386 |
| Concomitant therapy[d] | 0.70 | 0.21-2.14 | 0.550 |
| Treatment administration[c] | 0.50 | 0.14-1.72 | 0.269 |
| Diagnosis[b] | 1.40 | 0.49-4.03 | 0.515 |

**Per protocol, logistic regression, univariate**

| Variables | OR | 95% CI | p-value |
|---|---|---|---|
| Group[a] | 0.8 | 0.3-1.96 | 0.67 |
| Concomitant therapy[d] | 0.72 | 0.21- 2.13 | 0.57 |
| Treatment administration[c] | 0.39 | 0.21-0.66 | 0.001 |
| Diagnosis[b] | 1.24 | 0.41-3.48 | 0.515 |

**Per protocol, logistic regression, multivariate**

| Variable | OR | 95% CI | p-value |
|---|---|---|---|
| Group[a] | 0.70 | 0.25-1.93 | 0.488 |
| Concomitant therapy[d] | 0.58 | 0.16-1.86 | 0.380 |
| Treatment administration[c] | 0.54 | 0.13-1.82 | 0.343 |
| Diagnosis[b] | 1.37 | 0.44-4.12 | 0.574 |
